# Risk of depression, anxiety, and stress among the Saudi general population during the COVID-19 pandemic

**DOI:** 10.1186/s40359-022-01010-4

**Published:** 2022-12-14

**Authors:** Bandar Alsaif, Fahad D. Algahtani, Mohamed Ali Alzain, Rafat Zrieq, Badr K. Aldhmadi, Badr Alnasser, Sehar-un-Nisa Hassan

**Affiliations:** 1 Department of Public Health, College of Public Health and Health Informatics, University of Ha’il, PO Box 2440, Ha’il, 81451 Saudi Arabia; 2 Department of Health Management, College of Public Health and Health Informatics, University of Ha’il, PO Box 2440, Ha’il, 81451 Saudi Arabia; 3 Applied Science Research Center, Applied Science Private University, Amman, Jordan

**Keywords:** COVID-19 pandemic, Mental health, Depression, Anxiety, Stress, Risk factors

## Abstract

**Background:**

Promoting mental health and wellness is crucial for healthy communities. This study aims to assess the vulnerability of experiencing psychological reactions such as depression, anxiety, and stress within the general population in Saudi Arabia during the COVID-19 pandemic.

**Methods:**

A cross-sectional online survey was completed by 754 participants recruited from thirteen regions of Saudi Arabia. The information on background variables was obtained by using a set of close-ended questions. The DASS-21, a screening tool was used to assess the risk of depression, anxiety, and stress. Pie-chart displayed the levels of risk to experience psychological reactions. The Chi-square test and Adjusted Odd Ratios (AOR) reported the risk factors associated with experiencing psychological reactions in the study population.

**Results:**

The vulnerability to mild to extremely severe levels of stress, anxiety and depression were (36.5%), (34.9%), and (43.5%), respectively. Findings demonstrated that females were at higher risk to experience anxiety (AOR = 1.56; 95% CI 1.11–2.18) and stress (AOR = 1.48; 95% CI 1.10–2.10) as compared to males. Unmarried individuals had higher vulnerability for anxiety (AOR = 1.60; 95% CI 1.04–2.44) and depression (AOR = 1.55; 95% CI 1.02–2.37) as compared to married individuals. Those who lost their job during the pandemic had a higher risk to experience anxiety (AOR = 2.02; 95% CI 1.10–3.74) and depression (AOR = 2.01: 95% CI 1.09–3.87) as compared to those who remain employed. Diagnosis with COVID-19 was associated with anxiety (AOR = 2.40; 95% CI 1.27–4.52) and stress (AOR = 2. 0; 95% CI 1.10–3.69). Participants with chronic medical conditions were almost twice at risk to experience stress (AOR = 2.0; 95% CI 1.38–2.87) depression (AOR = 2.14; 95% CI 1.53–2.99) and anxiety (AOR = 2.59; 95% CI 1.78–3.78) as compared to those without such conditions.

**Conclusions:**

Findings imply the allocation of adequate psychological resources to prevent long-term psychological repercussions in at-risk populations such as females, unmarried individuals, those who lost their jobs, diagnosed with COVID-19 and those with chronic medical conditions.

## Introduction

Psychological impacts of the COVID-19 pandemic have been shown to be common in the general population by a systematic review and meta-analysis of studies conducted during the early period of the pandemic [1]. The symptoms of stress, anxiety and depression experienced by the general population during the pandemic are attributed to high levels of uncertainty and frustration [2]. As a disease-containment management strategy, lockdown and social distancing measures have been implemented in several countries, including the Kingdom of Saudi Arabia (KSA), and this strategy may have caused feelings of loneliness and distress in the general population [3, 4]. The impact of pandemic and quarantine measures on community mental health was reported during and after the outbreak of severe acute respiratory syndrome (SARS) in 2003 [5]. In the context of the COVID-19 pandemic, infodemic factors and high rates of morbidity and mortality seen within a few months of the epidemic outbreak in various parts of the world increased the risk to develop generalized stress and pervasive anxiety. Moreover, higher levels of state anxiety in comparison to trait anxiety are attributable to the COVID-19 pandemic situation [6].

Given that the COVID-19 outbreak has impacted individuals and societies in several ways, it is imperative to explore the demographic and social factors that may associate with the risk to experience psychological distress. The literature has demonstrated that high rates of fear due to infection, frustration, pervasive anxiety, and feelings of loneliness during lockdown have a negative impact on subjective well-being. Feelings of stress, anxiety and depression are also experienced by general populations due to their own vulnerability to infection as well as worry and concerns for loved ones [5–7]. The vulnerability to the psychological repercussions of the pandemic is likely to vary by the demographic, social and economic context of individuals [8]. Previous research showed that age, sex, and comorbid physical and mental illnesses were significantly associated with stress during the COVID-19 pandemic [9]. A study from the general population in Spain reported younger individuals with chronic diseases reported more symptoms of stress, anxiety and depression than the rest of the population [10].

The literature on the psychological impacts of the COVID-19 pandemic provides estimates of the rates of psychological distress and factors that may increase the vulnerability of some segments of the population to poor mental health [11]. Nonetheless, the findings from these studies need to be further validated and explained in view of the unique social, economic and cultural context of local communities in various parts of the world. Massive reporting on the impacts of pandemics, which undoubtedly disrupt the normal living conditions of people in various ways, can lead to desensitization at both the individual and community levels [12]. To avoid underdiagnoses or overdiagnoses of psychological morbidity in the general population, the need for more research is unprecedented.

The current research aims to assess the risk to develop stress, anxiety and depression symptoms. Several tools exist to assess psychological responses such as Depression Anxiety and Stress Scale (DASS-21) [13], Beck Depression Inventory [14], Hamilton Anxiety and Depression Scale [15], Hospital Anxiety and Depression Scale and Zung Self-rating Anxiety Scale [16]. In one study, authors developed the COVID-19 Peritraumatic Distress Index to measure traumatic stress experienced by the general population and healthcare workers in Saudi Arabia [17]. We used DASS-21 to measure the risk to develop depression, anxiety and stress in the general population because this tool is a robust screening tool in terms of psychometric properties and is available in the Arabic language. Moreover, it was employed in other multi-country studies to assess the symptoms of depression, anxiety and stress during the pandemic thus it was possible to make the appropriate comparisons.

Studying the psychological impacts of the COVID-19 pandemic on communities has been emphasized in literature because tapping the psychological experiences of people during the pandemic and lockdown periods will help to determine the mental healthcare needs of vulnerable populations [18]. Such studies contribute to determining the overall risk and redirecting community health resources to mitigate negative impacts. This study was designed in the same spirit to assess the risk of developing psychological reactions among the population in Saudi Arabia during the COVID-19 pandemic period. The government of Saudi Arabia is committed to improving the health and well-being of people in Saudi Arabia under Vision 2030 [19]. This vision was launched, as a roadmap, to make reforms in various sectors including health and to enhance the quality of life of individuals in the Kingdom. One of the objectives set for achieving population health focuses on the prevention of health risks. It aims at developing policies and programs for preventive public health to reduce exposure to disease, and the management of health crises pertaining to both communicable and non-communicable diseases, including epidemics and natural disasters. During the COVID-19 pandemic, a variety of predisposing and precipitating factors may increase or decrease exposure to psychological distress. For instance, social and economic factors such as gender, age, employment, and health conditions might relate to the risk of experiencing psychological reactions during the COVID-19 pandemic. Thus it is important to assess the relationship of such factors with psychological vulnerability at the country, regional and global levels. These studies are important to generate cumulative evidence for devising health policies and community health programs. The current study, therefore, aimed at determining the risk of stress, depression and anxiety and how this vulnerability differs across gender, age, education, marital status, nationality, diagnosis with the COVID-19 infection and comorbid health conditions in the general population in Saudi Arabia. Keeping in view the COVID-19 pandemic appeared as a national, regional and global health crisis, findings from such studies have wide generalizability. The determination of the risk to experience psychological responses during the pandemic in the Saudi population will provide useful insights to devise preventive interventions for mental health and wellness that aligns with the above-mentioned objective of the 2030 vision of the Kingdom of Saudi Arabia.

## Materials and methods

### Participants & procedure

Data were collected through an online survey, and responses were obtained from people living in thirteen KSA regions. The minimum sample size was 732, as calculated by using the formula: n = z^2^P (1−P)/d^2^ [20, 21] based on the assumption that the proportion of the psychological impact of the outbreak as moderate or severe in the previous study is 23.6% [22]. With a 95% confidence interval (CI), 0.05 acceptable sample error, z is selected critical value at 95% CI equals 1.96, and a design effect of 1.9 as we used non-probability sampling [21]. The data collection was completed by using online means due to the implementation of social distancing restrictions during the pandemic period. The target population for this study was the general population residing in thirteen regions of Saudi Arabia. The authors of this project have used professional links both at educational and healthcare institutions in thirteen regions of Saudi Arabia to recruit focal persons who supported in data collection from all over Saudi Arabia. A variety of social media tools including WhatsApp, Twitter, Snapchat, and institutional platforms were used to send study invites to the target population. The link included the invitation to participate in the study, informed consent, and inclusion criterion screening, followed by a study questionnaire. The participants had a choice to complete either English or Arabic versions of the questionnaire. The data collection was continued till the acquired sample size was achieved. It is not possible to determine the response rate because the link was shared on multiple platforms and a total of 754 respondents completed the survey link. There was no missing data which is most likely attributed to the use of electronic survey forms. In case of missing responses, the system intimates the participants to complete responses before final submission. All participants read and sign the electronic informed consent before proceeding to make responses to the study questionnaire. The research was approved by the Ethical Review Committee at the University of Hail.

### Study variables and instruments

*Background information* The participants provided demographic information, including sex, age, education, marital status, region of living and exposure to COVID-19 infections.

*Depression, anxiety and stress* An Arabic version of the Depression Anxiety Stress Scale (DASS-21) was used to screen the symptoms of depression, anxiety and stress in participants [13]. The DASS-21 is a shortened version of the full DASS and comprises of 21 items distributed among three subscales of depression, anxiety, and stress. Each subscale contains seven items rated on a 4-point Likert scale ranging from 0 (did not apply to me at all) to 3 (applied to me very much) and determines the levels of depression, anxiety, and stress. The cutoff scores are determined on the subscales of depression, anxiety, and stress to categorize the severity of symptoms from mild to extremely severe. On the depression subscale, scores in the range of 0–4 were categorized as no risk, scores in the range of 5–6 were categorized as mild, scores in the range of 7–10 were categorized as moderate, scores in the range of 11–13 were categorized as severe, and scores > 14 were categorized as extremely severe. On the anxiety subscale, scores in the range of 0–3 were categorized as no risk, scores in the range of 4–5 were categorized as mild, scores in the range of 6–7 were categorized as moderate, scores in the range of 8–9) were categorized as severe, and scores > 10 were categorized as extremely severe. On the stress subscale, scores in the range of 0–7 were categorized as no risk for stress, scores in the range of 8–9 were categorized as mild, scores in the range of 10–12 were categorized as moderate, scores in the range of 13–16 were categorized as severe, and scores > 17 were categorized as extremely severe.

In our study, the DASS-21 scale demonstrated adequate internal consistency with a Cronbach’s alpha value of α = 0.94 and an internal reliability coefficient for the depression (α = 0.88), anxiety (α = 0.81), and stress (α = 0.89) subscales.

### Data analysis

IBM SPSS Statistics version 24 was used to analyze the data. Descriptive statistics (mean and percentage values) were computed to describe the basic characteristics of the study variables. For analysis, we classified the psychological responses as binary outcomes (no risk vs at risk); this was carried out by separating those who had scores in the category of ‘no risk’ from those who had either ‘mild’, ‘moderate’, ‘severe’ and ‘extremely severe’ levels of risk. Subsequently, the chi-square test was applied to test the significance of differences across subcategories of independent variables. Binary logistic regression analysis examined the predictive nature of independent variables. Adjusted odds ratios (AORs) with a 95% CI were used to present the logistic regression results; significance was determined at p < 0.05. All results were adjusted for the following factors (age, gender, education level, marital status, chronic disease status, loss of job during the pandemic, and those diagnosed with COVID-19 or have a family/ friend diagnosed with COVID-19.

## Results

In this study, a total of 754 respondents participated in the study. Among these 54.1% of the participants were males, and 45.9% were females. The mean age was 36 ± 10.9 years, ranging from 18 to 64 years. All the participants were literate; more than half (53%) had a high school education. Most of the participants were married (67%) and lived with their family members (91%). More than two-thirds (74.1%) were Saudi nationals. Nearly 7% of the participants were diagnosed with COVID-19, and 20% had either a family member or a friend diagnosed with COVID-19. (Table 1).

**Table 1:** Demographic data of study participants (N = 754)

Variables	n	%
*Sex*		
Males	408	54.1
Females	346	45.9
*Age in years*		
18–25 years	146	19.4
26–35 years	222	29.4
36–45 years	257	34.1
46–55 years	102	13.5
56–65 years	27	3.6
*Education*		
Middle school	10	1.3
High school	89	11
College/University	401	53.2
Postgraduate	253	33.6
*Marital status*		
Currently not married	246	32.6
Currently married	508	67.6
*Nationality*		
Saudi citizen	559	74.1
Resident	195	25.8
*Lost job during the COVID-19 pandemic*		
Yes	51	6.8
No	703	93.2
*Diagnosed with COVID-19 infection*		
Neither tested nor diagnosed	548	72.7
Diagnosed as positive	52	6.9
Family member/Friend diagnosed as positive	154	20.4
*Any chronic medical conditions*		
Yes	195	25.9
No	559	74.1

**Fig. 1:**
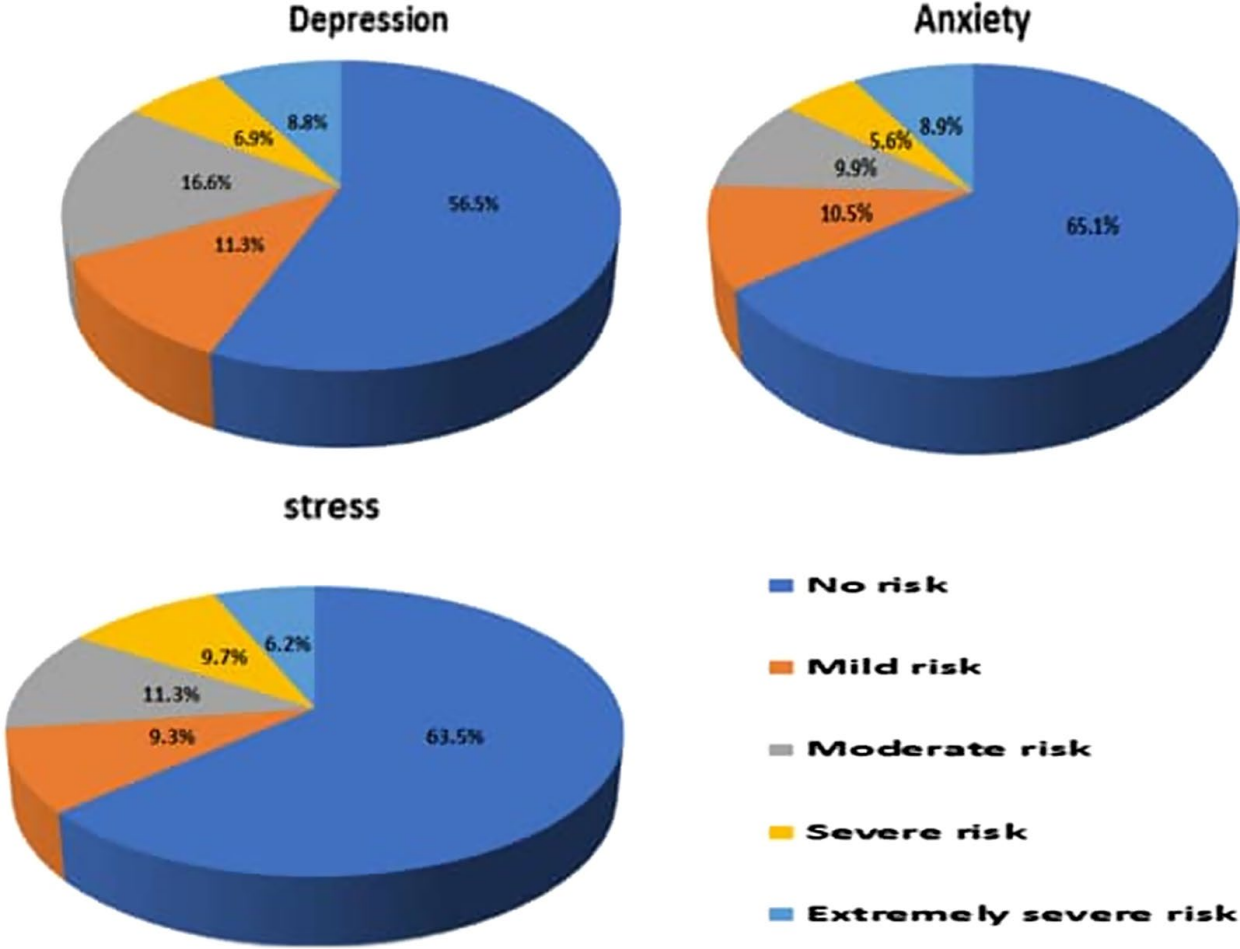
Risk of depression, anxiety and stress in the Saudi population during the COVID-19 pandemic in Saudi Arabia

Figure 1. presents the proportion of the risk of depression was found to be 43.5%, ranging from mild to extremely severe risk (11.3–8.8%). The proportion of the risk of anxiety was found to be 34.8%, ranging from mild to extremely severe risk (10.5–8.9%). In addition, the proportion of the risk of stress was found to be 36.5%, ranging from mild to extremely severe risk (9.3–6.2%).

Table 2 shows the bivariate association of demographic factors with the risk of depression, anxiety and stress. Findings show that individuals who were younger, currently not married, lost their job during the pandemic, or had a chronic disease were at higher risk of depression, anxiety, and stress than other groups.

**Table 2:** The association of demographic factors with risk to experience psychological responses during the COVID-19 pandemic in Saudi Arabia (N = 754)

Study variables	Depression		Anxiety		Stress	
	No risk n(%)	At risk n(%)	No risk n(%)	At risk n(%)	No risk n(%)	At risk n(%)
*Gender*						
Female	178(51.4)	168(48.6)**	205(59.2)	141(40.8)**	200(57.8)	146(42.2)**
Male	248(60.8)	160(39.2)	286(70.1)	122(29.9)	279(68.4)	129(31.6)
*Age in years*						
18–25	66(45.2)	80(54.8)***	68(46.6)	78(53.4)***	70(47.9)	76(52.1)***
26–35	97(43.7)	125(56.3)	135(60.8)	87(39.2)	130(58.6)	92(41.4)
36–45	175(68.1)	82(31.9)	199(77.4)	58(22.6)	188(73.2)	69(26.8)
46–55	66(64.7)	36(35.3)	70(68.6)	32(31.4)	71(69.6)	31(30.4)
56–65	22(81.5)	5(18.5)	19(70.4)	8(29.6)	20(74.1)	7(25.9)
*Education*						
Middle school	6(60.0)	4(40.0)^**ns**^	4(40.0)	5(60.0)**	6(60.0)	4(40.0)^**ns**^
High school	52(58.4)	37(41.6)	51(57.4)	38(42.6)	54(60.7)	35(39.3)
College/University	221(55.0)	181(45.0)	251(62.4)	151(37.6)	243(60.4)	159(39.6)
Postgraduate	147(58.1)	106(41.9)	185(73.1)	68(26.9)	176(69.6)	77(30.4)
*Marital status*						
Currently not married	108(43.9)	138(56.1)***	125 (50.8)	121 (49.2)***	125 (50.8)	121 (49.2)***
Currently married	318(62.6)	190 (37.4)	366 (72.0)	142 (28.0)	354 (69.7)	154 (30.3)
*Nationality*						
Saudi	320(57.2)	239(42.8)^**ns**^	353(63.2)	206(36.8)^**ns**^	342(61.2)	217(38.8)^**ns**^
Non-Saudi	106(54.4)	89(45.7)	138(70.8)	57(29.2)	137(70.3)	58(29.7)
*Lost job*						
Yes	18(35.1)	33(64.7)**	23(45.1)	28(54.9)**	25(49.0)	25(49.0)**
No	408(58.1)	295(42.1)	468(66.6)	235(33.4)	454(64.6)	249(35.4)
*Chronic health issues*						
Yes	90(46.2)	105(53.8)**	101(51.8)	94(48.2)***	105(53.8)	90(46.2)*
No	336(60.1)	223(39.9)	390(69.8)	169(30.2)	374(66.9)	185(33.1)
*Diagnosed with COVID-19*						
No	318(58.0)	230(42.1)^**ns**^	375(68.4)	173(36.1)**	367(67.0)	181(33.0)**
Yes	32(61.5)	20(38.5)	28(53.8)	24(46.2)	28(53.4)	24(46.2)
Family/friends	76(49.4)	78(50.6)	88(57.5)	66(42.9)	84(54.5)	70(45.5)

p value significance: ^ns^ > 0.05; * < 0.05; ** < 0.01 *** < 0.001

The logistic regression analysis was applied after checking the assumption of the multiple logistic regression. All models were adjusted for the factors (age, gender, education level, marital status, chronic disease status, loss of job during the pandemic, and those diagnosed with COVID-19 infection, those who have family/friends diagnosed with COVID-19) (Table 3). Findings showed that models were statistically significant in the anxiety, depression and stress risk χ^2^ = 91.84, χ^2^ = 97.898 and χ^2^ = 67.202 respectively p < 0.001, respectively and correctly classified 70–66% of those at-risk of having anxiety, depression and stress.

**Table 3:** Logistic regression to determine the risk of psychological reactions during the COVID-19 pandemic in Saudi Arabia (N = 754)

Variables	Depression		Anxiety		Stress	
	OR(95% CI)	AOR(95% CI)	OR(95% CI)	AOR(95% CI)	AOR(95% CI)	AOR(95% CI)
*Gender*						
Female	1.46(1.09–1.96)*	1.32(0.96–1.82)^ns^	1.61(1.19–2.18)**	1.56(1.11–2.18) **	1.58 (1.17–2.13)**	1.48 (1.10–2.10)*
Male	Reference	Reference	Reference	Reference	Reference	Reference
*Age group*						
18–25 years	5.33(1.92–14.85)**	5.92(1.93–18.11)**	2.72(1.12–6.62)*	2.53(0.922–6.92)^ns^	3.10(1.24–7.78)*	2.76(0.98–7.71)^ns^
26–35 years	5.67(2.1–15.52)**	8.29(2.86–23.9)***	1.53(0.64–3.65)^ns^	2.33(0.91–6.02)^ns^	2.02(0.82–4.98)^ns^	2.59(0.98–6.85)^ns^
36–45 years	2.06(0.75–5.64)^ns^	2.97(1.03–8.53)*	0.69(0.28–1.66)^ns^	1.10(0.43–2.84)^ns^	1.04(0.42–2.59)^ns^	1.47(0.55–3.86)^ns^
46–55 years	2.40(0.84–6.88)^ns^	3.05(1.01–9.20)*	1.08(0.43–2.74)^ns^	1.65(0.60–4.49)^ns^	1.25(0.48–3.25)^ns^	1.81(0.65–5.04)^ns^
55–65 years	Reference	Reference	Reference	Reference	Reference	Reference
*Nationality*						
Non-Saudi	1.12(0.81–1.56)^ns^	1.70(1.16–2.55)**	1.41(.99–2.01)^ns^	1.22(0.79–1.89)^ns^	1.49(1.05–2.12)*	0.98(0.65–1.48)^ns^
Saudi	Reference	Reference	Reference	Reference	Reference	Reference
*Education*						
Middle school	0.92(0.25–3.39)^ns^	1.21(0.30–4.83)^ns^	4.08(1.11–14.9)*	3.88(0.94–16.03)^ns^	1.52(0.41–5.52)	1.30(0.32–5.20)^ns^
High school	0.98(0.60–1.61)^ns^	0.93(0.51–1.70)^ns^	2.02(1.25–3.35)**	1.57(0.85–2.89)^ns^	1.48(0.89–2.44)	1.06(0.58–1.93)^ns^
University	1.13(0.82–1.56)^ns^	1.01(0.69–1.51)^ns^	1.63(1.16–2.31)**	1.3(0.86–1.94)^ns^	1.49(1.07–2.08)*	1.14(0.77–1.68)^ns^
Postgraduate	Reference	Reference	Reference	Reference	Reference	Reference
*Marital status*						
Currently not married	2.13(1.57–2.91)***	1.55(1.02–2.37)*	2.49(1.81–3.42)***	1.60(1.04–2.44)*	2.22(1.62–3.04)***	1.50(0.99–2.27)^ns^
Currently married	Reference	Reference	Reference	Reference	Reference	Reference
*Loss of job*						
Yes	2.53(1.40–4.59)**	2.01(1.09–3.87)*	2.42(1.36–4.30)**	2.02(1.10–3.74)*	1.89(1.07–3.34)*	1.60(0.85–2.85)^ns^
No	Reference	Reference	Reference	Reference	Reference	Reference
*Chronic health issues*						
Yes	1.75(1.26–2.44)^**^	2.3(1.59–3.32)***	2.14(1.53–2.99)**	2.59(1.78–3.78)***	1.73(1.24–2.41)**	2.0(1.38–2.87) ***
No	Reference	Reference	Reference	Reference	Reference	Reference
*Diagnosed with COVID-19*						
Yes	0.86(0.48–1.54)^ns^	1.01(0.54–1.88)^ns^	1.85(1.04–3.29)*	2.40(1.27–4.52)**	1.73(0.97–3.04)*	2.0(1.10–3.69)*
Friends/family members	1.41(.991–2.03)*	1.40(0.95–2.08)^ns^	1.62(1.12–2.34)**	1.54(1.03–2.30)*	1.69(1.17–2.43)**	1.63(1.10–2.40)*
No	Reference	Reference	Reference	Reference	Reference	Reference

*OR* odds ratio; *CI* confident interval; *AOR* adjusted odds ratio

Significance: ^ns^ > 0.05; * < 0.05; ** < 0.01 *** < 0.001;

The finding demonstrates that females were almost ≥ 1.5 times more likely to experience anxiety (AOR = 1.56; 95% CI 1.11–2.18) than males. Unmarried participants were more than one and a half times at risk to develop anxiety (AOR = 1.60; 95% CI 1.04–2.44). Participants who lost their job during the pandemic were prone to anxiety (AOR = 2.02; 95% CI 1.10–3.74). Participants with chronic medical conditions were almost two times at risk to have anxiety (AOR = 2.59; 95% 1.78–3.78). Lastly, findings showed that participants who were themselves diagnosed with the COVID-19 or those who had friends or/and family members diagnosed with COVID-19 were at two times more risk to experience anxiety (AOR = 2.40; 95% CI 1.27–4.52) and (AOR = 1.54; 95% CI 1.03–2.30), respectively.

Younger participants were at greater risk of depression compared with elder participants. Participants of age (18–25 years) were almost five times (AOR=5.92; 95% CI 1.93–18.11), and participants of age (26–35 years) were more than eight times (AOR = 8.29; 95% CI 2.86–23.9) at risk to experience symptoms of depression. The risk to have depression symptoms for participants of age (36–45 years) and (46-55 years) was threefold (AOR=2.97; 95% CI 1.03-8.53) and (AOR = 3.65; 95% CI 1.01–9.20), respectively as compared to the reference group. Unmarried participants were more than one and a half times at risk of depression (AOR = 1.55; 95% CI 1.02–2.37). Non-Saudi citizens were almost twice times (AOR = 1.70; 95% CI 1.16–2.55) more likely to experience depression than Saudi citizens. Participants who lost their job during the pandemic were at higher risk of depression (AOR = 2.01: 95% CI 1.09–3.87). Participants with chronic medical conditions were almost twice as likely to experience depression (AOR = 2.14; 95% CI 1.53–2.99).

The finding demonstrates that females were almost ≥ 1.5 times more likely to experience stress (AOR = 1.48; 95% CI 1.10–2.10) than males**.** Participants with chronic medical conditions were twice as likely to experience stress (AOR = 2.0; 95% CI 1.38–2.87). The findings demonstrated that participants who diagnosed or had friends or/and family members diagnosed or with COVID-19 were at increased risk of stress (AOR = 2.0; 95% CI 1.10–3.69) and (AOR = 1.63; 95% CI 1.10–2.40), respectively compared to those who not diagnosed with COVID-19 infection.

## Discussion

This study assessed the risk of depression, anxiety and stress in different segments of the population in Saudi Arabia during the COVID-19 outbreak. The findings show that nearly more than one-third of the respondents in this survey were at risk to develop stress, anxiety, or depression. To be more precise, the risk to experience mild to severe depression, anxiety and stress were 43.5%, 34.9% and 36.5%. The rates of risk to develop depression, anxiety and stress are slightly elevated than a prior study which collected data soon after the outbreak of the pandemic in Saudi Arabia which reported mild to severe levels of depression (40.9%), anxiety (29.9%), and stress (30.4%) [22]. The slight elevation could be attributed to the impact of the prolonged lockdown that was strictly implemented in Saudi Arabia, the increased rates of morbidity and mortality over the passage of time could have increased the risk to experience psychological reactions. The findings are comparable with other cross-sectional studies conducted during the pandemic period in other developed countries such as China, the UK, and Australia. The rates of moderate to severe levels of depression and anxiety in the general populations of these countries were between 37 and 62% [11, 23, 24]. Findings from this study demonstrated the vulnerability of certain sub-groups of the population such as women, young individuals, those who lost jobs, have direct and indirect exposure to the COVID-19 infection and those with chronic health conditions. Keeping in view the COVID-19 pandemic appeared as a global health crisis thus findings are applicable and found comparable with other studies. This study has a significant impact because findings provide insight to develop psycho-social interventions that generate large-scale improvements in health and well-being for at-risk populations and encourage the governing bodies to take measures for improving psychological health during and after the crisis.

The findings from our study demonstrate those female respondents were at increased risk to have anxiety and stress during the COVID-19 pandemic thus validating the prior evidence which showed that females were approximately five times more likely to suffer from posttraumatic stress and approximately two times more likely to have symptoms of depression, anxiety and stress during the pandemic [22]. In general, previous studies have also reported women’s vulnerability to psychological morbidity in comparison to men [25, 26]. Studies from Middle East countries have reported women’s increased vulnerability to common psychological problems such as depression, anxiety, and stress compared to males [27, 28]. There could be certain reasons for the high probability of stress and anxiety among women, such as women of reproductive age are usually more frequent consumers of maternal and child healthcare services and recreational resources in the community. The restricted access during the lockdown might have generated psychological responses. These findings suggest that community mental health programs be adapted to online modes and expanded to provide counselling and support services to at-risk groups of women. Women who are seeking services should be screened for psychological symptoms and provided with appropriate referral services to prevent the prolongation or adverse outcomes on their health. Moreover, mental health is not restricted to the presence or absence of mental disorders but to foster healthy communities it is important to promote mental well-being. The vulnerability to poor mental health in one segment of the population may add risk to another group, for instance, women’s susceptibility to experiencing anxiety and stress not only compromise their well-being but increase the risk for their children and have negative implications on family functioning. The evidence generated from this study has significance to gain public attention and political focus to address the psychosocial needs of at-risk individuals in societies.

Recently, the focus of governing authorities on Saudization and women’s empowerment has allowed more women of this age to enter the KSA job market [29]. Lockdown during the COVID-19 pandemic has restricted women from social and economic activities, which are already meagre in patriarchal societies. Exposure to various factors, such as economic dependence, low social support and poor environmental QOL, may increase the risk of stress and anxiety symptoms during the pandemic for women. Findings imply that women’s health programs should integrate psycho-social guidance and support to enhance their coping strategies and minimize the impact of these difficulties on their psychological health.

Our study findings demonstrate that the risk to experience symptoms of depression was higher in the younger population. These findings are in line with a study which collected data from Middle East countries that indicate an increased rate of stress disorders among youth during the COVID-19 pandemic [30]. The higher risk of stress symptoms in this age group is explainable in the context of Saudi youth culture, as they are more engaged in social, economic and educational activities. The strict and prolonged implementation of lockdowns in various KSA cities affected these activities and was likely a source of stress and anxiety for young adults [31]. The study findings imply the need for more youth support programs to enhance resilience and positive coping among this segment of the population. Findings also showed that non-Saudi residents in Saudi Arabia had a higher risk of depression. This could be due to the fact a large section of residents is living in Saudi Arabia for sake of employment, away from their immediate family, and they have limited access to social and emotional support. The pandemic has influenced the lives of individuals in various regions of the world and the feelings of loneliness and sadness are likely to elevate due to the high rates of morbidity and mortality associated with the pandemic around the globe. Despite, lockdowns and social distancing being effective measures to control the spread of infection, the psychological impacts of such measures on various segments of the community shouldn’t be overlooked.

In addition, the current findings demonstrate the vulnerability of those diagnosed with COVID-19 to stress, anxiety, and depression symptoms. These findings align with previous evidence [32, 33]. Moreover, the levels of stress and anxiety were significantly higher among people who reported living with a family member or friend diagnosed with COVID-19. The feelings of distress, anxiety and stress are likely to be high due to their attachment to loved ones and concerns over their health [34]. These findings imply timely access to psycho-social support for individuals and families who were directly or indirectly exposed to the physical and mental burden of the COVID-19 infection.

Participants diagnosed with chronic health conditions were at significantly higher risk of depression, anxiety and stress. The common chronic health conditions in Saudi Arabia include hypertension, high cholesterol, ischemic heart disease, obesity, kidney disease and diabetes [35, 36]. Other studies have reported that women in Saudi Arabia are potentially at an increased risk of developing obesity, cardiovascular diseases and diabetes mellitus due to sedentary lifestyles and their dietary habits [37]. In our study, more than a quarter of the participants reported suffering from chronic medical conditions, and this rate is higher than the previously reported 18% rate among primary healthcare patients [28]. Among this group, 46–53% reported having symptoms of stress, anxiety or depression. This rate is alarmingly high and signifies the need to provide adequate care and resources to people with chronic health conditions because the risks of physical confinement during quarantine and complete lockdown affect them disproportionately [38]. In the later stages of the lockdown, the Ministry of Health in Saudi Arabia expanded digital solutions to support healthcare services and allowed people to walk and partake in physical activity during the lockdown period [39].

Some of the limitations of this study should be considered while interpreting findings. Firstly, the cross-sectional descriptive design was employed, and data was collected only once thus it is not possible to demonstrate that risks of depression, anxiety and stress are directly linked with the situation of the pandemic. However, the screening tool assesses the presence of these symptoms in the past week, and the study assessed the relationship of some relevant factors such as loss of job during the pandemic, diagnosis of the COVID-19 infection, having a friend or family member diagnosed with the COVID-19 with the risk to have depression, anxiety and stress. Secondly, the data was collected by employing an online self-report survey form, due to the implementation of social distancing thus, it is important to consider biases inherent in self-report measures while interpreting findings from this study. Thirdly, we recruited focal persons in all regions to reach out target population however, community members who do not use social media tools for communication and those who are not able to read or record their responses on electronic survey forms are excluded systematically. Lastly, the assessment of mental health symptoms was made by using the short form of DASS-21, which is not a replacement for a clinical interview or other diagnostic tools for the assessment of psychopathology. The current finding thus only indicates the risk to experience psychological reactions and should not be interpreted as a disorder or pathological condition.

Overall, the assessment of risk and identification of vulnerable sub-groups through this study provides useful insights to promote community mental health. Community health interventions should focus on mental wellness programs for at-risk populations. Expansion of telehealth services to increase access to healthcare for people with chronic medical conditions. The insights gained from this epidemiological study are helpful to determine the psychological impact of the pandemic on various segments of the population and the need for action. Expansion of existing resources and scaling up of community mental health programs will be effective to reduce the short-term and long-term psycho-social impacts of this crisis. These solutions will also contribute to the attainment of targets set for the 2030 vision for Saudi Arabia, which aims at offering a fulfilling and healthy life to people living in Saudi Arabia [19]. Promoting mental health is essential to develop resilience among individuals, families and communities thus enabling them to effectively deal with challenging situations. Future studies should adopt qualitative methods to gain deeper insight into necessary measures because early and efficient access to psychosocial support is crucial in building healthy and resilient communities.

## Conclusion

Psychological vulnerability during a crisis period such as the COVID-19 pandemic is expected. However, this study like other epidemiological studies provided useful insights for the adoption of precautionary measures. Findings imply the allocation of adequate psychological resources to prevent long-term psychological repercussions in at-risk populations such as females, young individuals, those who lost their jobs, diagnosed with COVID-19 and those with chronic medical conditions. Populations should be equipped with adequate internal resources and external support to deal with the psychological aftermaths of calamities, which are crucial to promote resilience and community health.

## Data Availability

The datasets used and/or analyzed during the current study are available from the corresponding author upon reasonable request.
